# Antibacterial Activity of 2-Picolyl-polypyridyl-Based Ruthenium (II/III) Complexes on Non-Drug-Resistant and Drug-Resistant Bacteria

**DOI:** 10.1155/2021/5563209

**Published:** 2021-05-20

**Authors:** James T. P. Matshwele, Sebusi Odisitse, Daphne Mapolelo, Melvin Leteane, Lebogang G. Julius, David O. Nkwe, Florence Nareetsile

**Affiliations:** ^1^Botswana International University of Science and Technology, Department of Chemistry and Forensic Sciences, Private Bag 16, Palapye, Botswana; ^2^Botho University, Department of Applied Sciences, PO Box, Gaborone 501564, Botswana; ^3^University of Botswana, Faculty of Science, Private Bag, Gaborone 0704, Botswana; ^4^Botswana International University of Science and Technology, Department of Biological Sciences and Biotechnology, Private Bag 16, Palapye, Botswana

## Abstract

A new hexadentate 2-picolyl-polypyridyl-based ligand (4, 4'-(butane-1, 4-diylbis(oxy))bis(N, N-bis(pyridin-2-ylmethyl)aniline)) (2BUT) **(1)** and its corresponding Ru(II/III) complexes were synthesized and characterized, followed by assessment of their possible bioactive properties towards drug-resistant and non-drug-resistant bacteria. Spectroscopic characterization of the ligand was done using proton NMR, FTIR, and ESI-MS, which showed that the ligand was successfully synthesized. The Ru(II/III) complexes were characterized by FTIR, UV/Vis, elemental analysis, proton NMR, ESI-MS, and magnetic susceptibility studies. The analysis of ESI-MS data of the complexes showed that they were successfully synthesized. Empirical formulae derived from elemental analysis of the complexes also indicated successful synthesis and relative purity of the complexes. The important functional groups of the ligands could be observed after complexation using FTIR. Magnetic susceptibility data and electronic spectra indicated that both complexes adopt a low spin configuration. The disc diffusion assay was used to test the compounds for antibiotic activity on two bacteria species and their drug-resistant counterparts. The compounds displayed antibiotic activity towards the two non-drug-resistant bacteria. As for the drug-resistant organisms, only [Ru_2_(2BUT)(DMF)_2_(DPA)_2_](BH_4_)_4_**3** and 2, 2-dipyridylamine inhibited the growth of MRSA. Gel electrophoresis DNA cleavage studies showed that the ligands had no DNA cleaving properties while all the complexes denatured the bacterial DNA. Therefore, the complexes may have DNA nuclease activity towards the bacterial genomic material.

## 1. Introduction

Ever since the success of cisplatin as an antitumor agent, other platinum group metal complexes have gained a lot of interest as potential bioactive agents, especially those of ruthenium [1–6]. Over the years, ruthenium complexes were observed to have bioactive properties as potential antimicrobial, anticancer, and antiviral agents [7–10]. Ruthenium does not have any specific biochemical role but is still not toxic in biological systems [1]. Ruthenium complexes have also gained much interest because of their special chemical properties such as having similar ligand exchange properties to Pt (II) complexes [2]. Therefore, some complexes of ruthenium would have similar bioefficacy to platinum complexes, although having lower cytotoxicity as opposed to platinum complexes [2]. Furthermore, ruthenium can access variable oxidation states (II, III, and IV). The interconversion potential associated with these ruthenium oxidation states is known to be relatively low. Thus, changing through these oxidation states in biological systems may not be difficult. Also, the low toxicity of ruthenium complexes has been observed to come from the inert Ru(III) complexes which have been shown to become active when reduced to Ru(II) by oxidative reductive biochemical environments such as cancer cell environments [11–13].

Various literature reports have demonstrated that ruthenium complexes bearing pyridyl moieties have potent antibacterial activities and this has been associated with variable oxidation states, the low toxicity of the ruthenium centre, and the ability of pyridinyl rings to penetrate membrane walls of bacteria. A few examples such as those with 1, 10-phenanthroline and guanine ligands are known to exhibit some antibacterial activity towards Gram-positive and Gram-negative bacterial strains. These complexes showed biological activity towards Gram-positive and Gram-negative bacterial strains. Their antibacterial activity against the multidrug-resistant (MDR) *Klebsiella pneumoniae* is reported to be even higher than that of commercially available antibiotics chloramphenicol and ciprofloxacin [3]. In another study by Gopinath et al., green chemistry synthesis of ruthenium nanoparticles (NPs) using leaf extracts of *Gloriosa superba* plant was carried out. These ruthenium-based green NPs exhibited some interesting broad-spectrum antibacterial activity towards both Gram-positive and Gram-negative bacteria [4]. Lam et al. developed some Ru(II) bis(2, 2′-bipyridyl) complexes with N-phenyl-substituted diazafluorenes and tested their bioactivity towards methicillin-resistant *Staphylococcus aureus* (MRSA) [5]. The lower minimum inhibitory concentration of their *cis*-dichlorobis(2, 2′-bipyridine)ruthenium compared to other complexes against the MRSA suggested that it had higher potency.

Our previous study [6] demonstrated that the polypyridyl ruthenium complexes exhibit antibacterial activity against two non-drug-resistant bacteria *S. aureus* and *Klebsiella pneumoniae.* The complexes showed broad-spectrum activity by inhibiting the growth of both Gram-negative and Gram-positive bacteria. In addition, all the ligands showed some activity towards the drug-resistant bacteria MRSA. However, no complexes had activity towards both MRSA and MDR *Klebsiella pneumoniae.* In that regard, we modified the ligands on the ruthenium centre with the hope of targeting the drug-resistant bacteria, and our findings are reported herein, where a dinuclear Ru(II) complex and a ligand displayed interesting antibacterial activity towards drug-resistant bacteria.

## 2. Experimental

### 2.1. Materials and Instrumentation

Chemicals and reagents used for synthesis and those for biological activity assays were purchased from Merck, Sigma-Aldrich, USA, and used without further purification. The neat film infrared spectroscopy of the compounds was recorded in the 4000–500 nm region using a PerkinElmer System 2000 FTIR spectrometer (PerkinElmer, USA). UV-Visible absorption spectra were recorded using a Shimadzu UV-31-1 PC scanning spectrophotometer (Shimadzu Corp, Japan) in 1 cm path length quartz cells at room temperature. HRESI-MS data were acquired using Bruker Daltonics micrOTOF-Q II ESI-Qq-TOF mass spectrometer (Bruker Daltonics, Germany). The acquisition parameters were as follows: HRESI-MS; drying gas was 50 L/h at 180°C drying gas temperature, the desolvation gas was 591 L/h at 300°C, and capillary voltages are 4.5 kV. ^1^H-NMR and spectra were acquired in 5 mm NMR tubes at 298 or 310 K by an Agilent 600 MHz spectrometer in the range 0–10 ppm. Solvents used were deuterated chloroform with the reference material TMS (Agilent Technologies, USA). The data were processed on MestReNova version 9.0.1–13254. Elemental analyses (CHNO) were performed with a model 240 PerkinElmer elemental analyser (PerkinElmer, USA).

### 2.2. Synthesis

**Figure 1:**
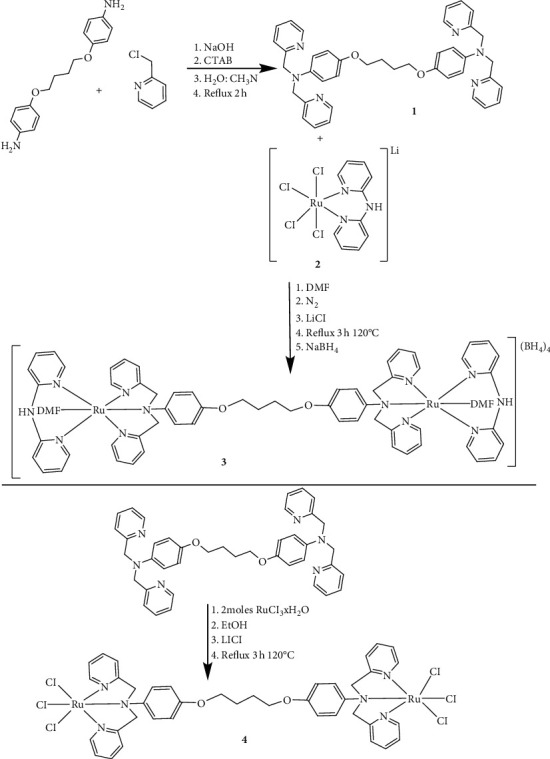
Synthetic routes for the ligand 2BUT **(1)** and ruthenium complexes: [Ru_2_(2BUT)(DMF)_2_(DPA)](BH_4_)_4_**(3)** and [Ru_2_(2BUT)(Cl)_6_] **(4)**.

2, 2-Dipyridylamine (DPA) ligand was purchased from Merck, Sigma-Aldrich, USA. The intermediate complex Li[Ru(Cl)_4_(DPA)] **2** was prepared according to the procedure in our previous study [6]. Briefly, the synthetic scheme for the synthesis of the ligand and all the complexes is shown in Figure 1.

#### 2.2.1. Preparation of 4, 4'-(Butane-1, 4-diylbis(oxy))bis(N, N-bis(pyridin-2-ylmethyl)aniline) (2BUT) **(1)**

4, 4'-(Butane-1, 4-diylbis(oxy))dianiline (160 mg, 0.60 mmol) and 2-chloromethylpyridine (200 mg, 1.2 mmol) were mixed in 10 mL (1 : 1) acetonitrile-to-water solvent mixture in a 50 mL round-bottom flask. Thereafter, half a drop of cetyl trimethyl ammonium bromide (CTAB) was added to assist in phase transfer when extracting the product. The above reagents were refluxed while 5 mL of sodium hydroxide (48 mg, 1.2 mmol) was added over a period of 30 min. The reaction was left to reflux for a further 2 h. The solution was then cooled and extracted with 30 mL portions of dichloromethane three times. The three portions of the extract were mixed and dried with anhydrous magnesium sulphate. Evaporation of the extract afforded a brown oil which solidified after several days in the air. Yield: 280 mg, 76.0 %. mp 145–147°C. IR (*υ*_max_/cm^−1^) (C-H) 2926, (C=C) 1508, (Ar-N) 1431, (C-O) 1230. ^1^H NMR (600 MHz, CDCl_3_, *δ*) 8.48 (m, 4H), 7.53 (m, 4H), 7.24 (m, 4H), 7.07 (m, 4H), 6.68–6.53 (dd, *J* = 8.6 Hz, 4H), 4.67 (s, 8H), 3.87–3.81 (t, 2H), 1.85–1.73 (q, 2H). HRESI-MS [M+H]^+^*m*/*z* 637.3412 (calcd for C_40_H_41_N_6_O_2_ 637.8075).

#### 2.2.2. Preparation of [Ru_2_(2BUT)(DMF)_2_(DPA)](BH_4_)_4_**(3)**

Lithium (tetrachloro)(dipyridylamine) ruthenate(III) (170 mg, 0.40 mmol), 4, 4'-(butane-1, 4-diylbis(oxy))bis(*N, N*-bis(pyridin-2-ylmethyl)aniline) (130 mg, 0.20 mmol), and lithium chloride (250 mg, 0.40 mmol) were refluxed in 10 mL dimethylformamide for 3 h under nitrogen atmosphere at 120°C. The blue-green solution slowly turned dark blue during the reaction. The solution was cooled and excess 30% sodium borohydride was added while stirring by means of a glass rod. The precipitate was extracted through liquid-liquid extraction to remove the excess dimethylformamide using the reaction mass with dichloromethane. The reddish amorphous powder was obtained. Yield: 160 mg, 62.0 %. IR (*υ*_max_/cm^−1^) (C-H) 2961 (C=C) 1504, (C-O) 1244, (Ar-N) 1434, (C=O_DMF_) 1668. ^1^H NMR (600 MHz, CDCl_3_) *d* 9.84–9.66 (m, 4H), 8.52 (m, 4H), 7.83 (m, 4H), 7.68 (m, 2H), 7.58–7.43 (m, 8H), 7.20–7.05 (m, 8H), 6.71 (m, 4H), 6.59 (m, 4H), 4.53 (s, 4H), 3.86 (m, 4H), 2.55 (m, 4H), 0.92–0.71 (m, 12H). UV-Vis (DMF; *λ*max (nm)): 262, 462, 532. HRESI-MS [M+2Na-H]+ *m*/*z* 1372.7879 (calcd for C_66_H_72_N_14_O_4_Ru_2_ 1373.3612). Anal. Found (%) for C_66_H_88_B_4_N_14_O_4_Ru_2_**•**0.9H_2_O: 56.89 %; H, 6.33 %; N, 13.59 %. Calcd (%) C, 56.50 %; H, 6.45 %; N, 13.98 %.

#### 2.2.3. Preparation of [Ru_2_(2BUT)(Cl)_6_] **(4)**

Ruthenium trichloride trihydrate (1000 mg, 4.8 mmol), 4, 4'-(butane-1, 4-diylbis(oxy))bis(*N, N*-bis(pyridin-2-ylmethyl)aniline) (1500 mg, 2.4 mmol), and lithium chloride (200 mg, 4.7 mmol) were all dissolved in 30 mL of absolute ethanol. The black mixture was refluxed for 3 h at 120°C and filtered while it was still hot. The deep purple precipitate formed during the reaction and was filtered immediately while hot by suction and washed with 3 × 30 mL of ethanol followed by 3 × 30 mL of diethyl ether. Yield: 1700 mg, 70.0 %. IR (*υ*_max_/cm^−1^) (H_2_O) 3403, (C-H) 2948, (C=C) 1508, (C-O) 1245, (Ar-N) 1435, (C=O_DMF_) 1949. ^1^H NMR (600 MHz, CDCl_3_) *δ* 7.46–8.06 (m, 24H), 3.76 (t, *J* = 6.4 Hz, 4H), 2.60 (s, 8H), 1.89–1.85 (m, 4H). UV-Vis (DMF; *λ*max (nm)): 297, 412, 604. HRESI-MS [M+K]^+^*m*/*z* 1104.5452 (calcd for C_41_H_43_Cl_6_N_6_O_2_Ru_2_ 1103.9332). Anal. Found (%) for C_41_H_43_Cl_6_N_6_O_2_Ru_2_**•**0.6H_2_O: 45.66 %; H, 3.70 %; N, 7.50 %. Calcd (%) C, 45.26 %; H, 3.90 %; N, 7.92 %.

### 2.3. Antimicrobial Studies

Disc diffusion assay and minimum inhibition concentration methods were followed as in our previous publication [6] and the National Committee for Clinical Laboratory Standards (NCCLS) as summarized in the literature [7]. All the sample organisms were clinical isolates that were kindly donated by the National Health Laboratory, Botswana, Gaborone. The minimal inhibitory concentration (MIC) was determined by the broth microdilution method. Six concentrations of each of the metal compounds were made in serial dilutions: (40, 20, 10, 5, 2.5, and 1.25) g/mL. Bacterial inocula, with an incubation time not over 24 h, were adjusted to the 0.5 McFarland standard and further diluted down to 5 × 10^5^ CFU/mL by double distilled water. For the determination of the MIC, serial dilution was made in MH broth to a final volume of 100 *μ*l in 96-well plates, and an aliquot of 100 *μ*l of the bacterial solution was added to each solution. The experiment was done using alamarBlue; this experiment shows a color change. In the color change, the MIC was observed as the first dilution without a color change from the blue solution. This was observed as the concentration of the metal compounds increases. The color change of the dye turns from blue to pink to indicate live microorganisms. The bioassays were performed in triplicate for accuracy. The bioassays were statistically evaluated using an ANOVA followed by *T*-test (*p* *<* 0.05).

The disc diffusion assay was briefly done as follows. Bacterial culture suspension was inoculated on Mueller-Hinton (MH) agar in 90 mm Petri dishes. The bacterial strains used were *S. aureus*, *K. pneumoniae*, MRSA, and MDR *K. pneumoniae*, and quality control bacteria such as *Staphylococcus aureus* (ATCC 25923), *Escherichia coli* (ATCC 25922), and *Klebsiella pneumonia* (ATCC 70063) were included in the experimental setup. Then, sterile Whatman filter paper discs, impregnated with test compounds (5 *μ*l at concentrations determined from MIC), were placed on the agar and then incubated at 35 ± 2°C for 18 h. For a negative control, a Petri dish containing only the MH culture medium was included. After the 18 h incubation, zones of inhibition including the diameter of the discs were measured. Inhibition zones above 7 mm in diameter were considered as positive results.

### 2.4. DNA Cleavage Assay

The method used was adopted as described before [6]. DNA binding ability of the test compounds towards the *S. aureus* bacterial DNA was determined by agarose gel electrophoresis to assess whether the compounds had any interaction with bacterial DNA. Ten microlitres of compounds that exhibited antimicrobial activities as determined in the MIC assay were mixed with 10 *μ*l of 53 *μ*g/mL of the *S. aureus* DNA in Tris-HCl/NaCl buffer solution and then incubated at 37 °C for 2 h. After incubation, the samples were run on 0.8% arose gel in Tris-acetic acid-EDTA buffer, at 60 V for 90 min. The gel was then stained with ethidium bromide and photographed under 254 nm UV light.

## 3. Results and Discussion

### 3.1. Synthesis of Compounds

The three new compounds, the ligand 2BUT **1** and complexes **3** and **4** shown in Figure 1, were prepared successfully in moderate amounts. Compound **1** was achieved by chemoselective alkylation of the oxygen nucleophile using 1, 4-bromobutane with 2 molar equivalent of 4-aminophenol to afford 4, 4'-(butane-1, 4-diylbis(oxy))dianiline intermediate ligand. This was done to block the competitive reaction between the oxygen and nitrogen nucleophiles of 4-aminophenol. The reaction path for the synthesis of the 2BUT ligand was proposed to follow the *S*_*N*_*2* mechanism. This was suggested because of the use of a strong base sodium hydroxide and the use of a primary benzylic/pyridyl halide 2-picolyl. The ligand was recovered as a brown oil that formed at a yield of 76%. The intermediate ligand 4, 4'-(butane-1, 4-diylbis (oxy))dianiline has four protons that were deprotonated by the strong base sodium hydroxide in order to attach the 2-picolinic arms. This new attachment of the 2-picolinic arms was observed spectroscopically, where in proton NMR there was an introduction of the aromatic multiplets coming from the 2-picolinic arms, while in FTIR there was disappearance of the (NH_2_) peaks in vibrational frequencies above 3000 cm^−1^. There was also a new peak of the aromatic nitrogen coming with the 2-picolinic arms observed at 1431.3 cm^−1^.

The reaction path for ruthenium complexes was proposed to follow the dissociative or the associative mechanism. This was because there was substitution of the ligands during the reaction. Also, the nitrogen-based ligand that was reacting with RuCl_3_•3H_2_O is a borderline base, and Ru(III) is also borderline. This made it possible for either the associative or dissociative mechanism to be favoured. So, in that regard, the pyridyl ligands were bound to the ruthenium centre to form the Ru(III) [8]. As for the Ru(II) complex, by using the reducing agent dimethylformamide as the solvent and excess pyridyl ligands, the ruthenium was reduced to the softer Ru(II) [8]. After many failed attempts to grow crystals, we resorted to using elemental analysis and other complementary data to characterize complexes **3** and **4** and their characterization is reported in the next section.

### 3.2. Characterization of the Ligands and Complexes

#### 3.2.1. Characterisation by Fourier Transform Infrared Spectroscopy

Characteristic vibrational frequencies of the available functional groups were assigned with FTIR. This was done to observe the stretching frequencies associated with important functional groups within the ligands and complexes. The important functional groups to look at in the new ligand as well as the complexes are shown in Table 1. The intermediate ligand 4, 4'-(butane-1, 4-diylbis (oxy))dianiline showed the amine (-NH_2_) vibration at the frequency of 3311–3387 cm^−1^, which disappeared upon attachment of the 2-picolyl moieties. The 2BUT ligand showed the following important functionalities that were still observed from the intermediate ligand: the aliphatic (C-H) vibration at a frequency of 2926 cm^−1^. The aromatic (C=C) vibrations at a frequency of 1508 cm^−1^ and the (C-O-C) vibration which is a sharp and strong peak at 1230 cm^−1^ were also observed. Lastly, we observed the appearance of the aromatic pyridyl nitrogen vibration (C=N) at 1431 cm^−1^. The commercially bought 2, 2-dipyridyl amine ligand fundamental peaks are also tabulated in Table 1, to compare with the new 2BUT ligand.

**Figure 2:**
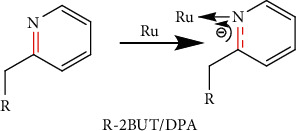
Ruthenium pulling electrons from the pyridyl nitrogen, therefore, reducing the electron density of the C=N bond together with the vibrational frequency of this bond as per Hooke's law.

The complexes FTIR data indicated that the complexes were formed. This was based on the binding nature of ruthenium to the ligands. According to Hooke's law, vibrational frequency is directly proportional to the strength of the spring. This means that the stronger the spring, the higher the vibrational frequency. Electron density may increase or decrease on the bonds due to complexation; thus, the vibrational frequency of the bonds may increase or decrease. There was an observed decrease in the vibrational frequency of the carbon-nitrogen bond of the pyridyl nitrogen, and this was assumed to be caused by the ruthenium inductively pulling electrons from the pyridyl nitrogen as depicted in Figure 2 [6]. All the other important functional groups coming from the ligands in the complexes were observed and tabulated in Table 1.

#### 3.2.2. Electronic Spectra and Magnetic Susceptibility

**Figure 3:**
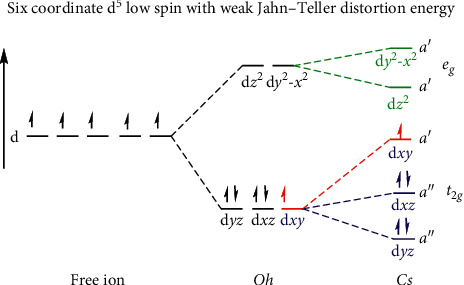
Suspected weak Jahn–Teller distortions on complex **4 ***Note*. The diagram is not to scale. The diagram demonstrates that the *e*_*g*_ orbitals transform under the a′ symmetry label while the *t*_2*g*_ orbitals transform under the a′, a″, and a″; this observation suggests the weak Jahn–Teller distortion. This explains why the observed effective magnetic moment was a bit lower than the expected magnetic moment which is because of the unpaired electron not having orbital contribution from interacting with the other orbitals, thus having no effect on the magnetic moment.

The electronic spectra and magnetic susceptibility studies were done to find the oxidation state and the spin of the complexes. Electronic transitions observed below 300 nm were assigned to ligand *π*-*π*^*∗*^ and other charge transfer transitions, especially that the pyridyl ligands have low-lying *π*-orbitals and the metallic ion has high energy unoccupied *d*-orbitals. However, all transitions observed above 400 nm were assigned to the *d-d* transitions. The Ru(III) complex **4** was expected to show at least four energetically observable *d-d* transitions according to its *d*^5^ Tanabe–Sugano diagram. The complex was assigned to be a low spin complex because of the ruthenium *4d* orbitals being large; this means that the pairing energy is most of the times smaller than the splitting parameter, thus resulting in low spin complexes with any ligand type. So, using a *d*^5^ Tanabe–Sugano diagram, complex **4** was assigned the *d-d* transitions 604 nm ^*2*^*T*_*2g*_ (*I*) *⟶* ^*2*^*T*_*2g*_,^*2*^*A*_*2g*_ (*I*) and *412 *nm ^*2*^*T*_*2g*_ (*I*) *⟶* ^*2*^*E*_*g*_(*I*) which were the energetically visible bands. These are summarized in Table 2, with molar extinction coefficients which also suggested that these were *d-d* transition bands. Furthermore, complex **4** had an experimental magnetic moment of 1.68 BM contrary to a spin-only calculated magnetic moment of 1.73 BM for a low spin *d*^*5*^ complex. From a low spin *d*^5^ Tanabe–Sugano diagram, it is observed that this complex has a ground term of *T*, meaning that it should have a spin-orbital contribution as this is true for complexes with *T* terms. Therefore, an effective magnetic moment of above 1.73 BM (calculated spin-only magnetic moment) was expected. However, this deviation can be explained by evoking the Jahn–Teller distortions as depicted in Figure 3.

The ligand used on this complex **4** had a pyridyl moiety which has many bonding interactions that made it above average in strength in the spectrochemical series. The *d-*orbitals of ruthenium interact with the following: the pyridyl *sp*^*2*^ hybrid lone pair of electrons in the aromatic nitrogen, the *π* electrons coming from aromatic conjugated *π* bonds, and the empty ring *π*-orbitals may also accept electrons from the metal orbitals too. These many orbital interactions were assumed to lead to some weak elongated Jahn–Teller distortions [9] on complex **4 ***d-*orbitals, which caused the effective magnetic moment to be lower than the spin-orbit and spin-only calculated magnetic moments. Figure 3 shows how these weak Jahn–Teller distortions may have contributed to this. The proposed structure of complex **4** is assumed to transform under the *C*_*s*_ point group. This point group's character table contained the totally symmetric A′ and the antisymmetric A″ symmetry elements. These symmetry elements showed that the *e*_*g*_ orbitals were totally symmetric and transformed under the A′ symmetry element, while the *t*_*2g*_ orbitals of this complex did not have the same symmetry and transformed under both A′ and A″. Two *t*_*2g*_ orbitals *d*_*yz*_ and *d*_*xz*_ were observed to transform under the A″ symmetry element. The A′ symmetry element contained the other *d*_*xy*_ orbital. From the character table, it showed that the *t*_*2g*_ orbitals that transform under the A″ were not degenerate but were both symmetrically similar. Thus, this suggests that these three *t*_*2g*_ orbitals are not on the same energy level, hence suggesting an elongated weak Jahn–Teller effect along the *z*-axis shown in Figure 3 [9]. This also meant that the electron on the *d*_*xy*_ orbital was unable to interact with the other electrons on the other *t*_*2g*_ orbitals to enforce spin-orbit contribution towards the effective magnetic moment of complex **4** [10].

The Ru(II) complex **3** was assumed to be a *d*^*6*^ low spin complex, and this was observed with an effective magnetic moment of 0 BM. However, according to a *d*^*6*^ Tanabe–Sugano diagram, at least 2 energetically allowed *d-d* transitions should be observed with one observable charge transfer band at 345 nm. And according to our observation, these transitions for complex **3** as summarized in Table 2 were at 532 nm ^*1*^*A*_*1g*_(*I*)* ⟶ *^1^*T*_*1g*_(*I*) and 462 nm ^*1*^*A*_*1g*_(*I*) *⟶* ^*1*^*T*_*2g*_(*I*). The calculated molar extinction coefficients of this complex also assume these transitions to be *d-d* transitions bands.

#### 3.2.3. Elemental Analysis of the Complexes

Elemental analysis was used to find the empirical formula and purity of the synthesized complexes. The elemental analytical data show that the complexes were successfully synthesized. However, both the complexes contain some water of crystallization in their formula. The Ru(II) complex contains at least 0.9 moles of water while Ru(III) contained at least 0.6 moles of water. The values for the experimental and calculated elemental analysis are shown in Table 3.

#### 3.2.4. Mass Spectrometric Characterization

A soft ionisation mass spectrometric technique, the electrospray ionization was used, and this was chosen due to the bulkiness of the test compounds. This type of technique has been successful in quantifying the masses of compounds with weak interactions because of its low-energy ionisation. From the data, it was observed that the experimental molecular ion of the test compounds was close to the calculated molecular masses. All the compounds were run under positive ion mode, thus giving positive molecular ion adducts. The ligand showed the formation of an adduct with one proton [M+H]^+^ to its experimental mass, while complex **3** showed adduct formation with two sodium ions and less of one proton to the experimental mass, [M+2Na-H]^+^. On the other hand, complex **4** showed an adduct [M+K]^+^ where there was one potassium ion to the experimental mass.  Table 4 shows these data.

#### 3.2.5. Proton NMR Characterization

One-dimensional proton NMR was used as a characterization tool to study the chemical shifts on the ligand proton NMR compared to that of the complexes. Considering the spectrum of the ligand 2BUT, the ligand molecule shows that there might be symmetry between the central (-CH_2_) groups on the central aliphatic diether chain, suggesting that the two sides of the molecule have similar chemical environments. Even the proton NMR spectral data showed only the chemistry of one side of the molecule. The 2BUT ligand has a *para*-substituted benzene aromatic ring that was expected to show a doublet of a doublet splitting pattern, which was observed at 6.52–6.68 ppm in the spectrum. The chemical shift data are displayed under Section 3.2. The 2BUT ligand showed eight observable protons from the aliphatic chain. Of these eight protons, four of each set showed the same chemical environment, therefore giving similar splitting which resulted in only two peaks. The four protons in the central *sp*^*2*^ carbons of 2BUT were coupled to the outer four protons within the central aliphatic chain leading to a quintet splitting pattern observed at 1.75–1.82 ppm. The other four outer protons which were on the carbon atoms directly bound to the oxygen had only two proton neighbours each. Thus, they showed a multiplicity of a triplet at 3.82–3.84 ppm. Furthermore, analysis of the 2BUT ligand 2-picolinic arms showed a total of four *sp*^*3*^ protons for each arm. These protons showed a multiplicity of a singlet since they are not split by any neighbouring protons. The splitting patterns of the aromatic pyridine moiety were similar for both rings as they shared a similar chemical environment. In addition, integration data also supported that there was symmetry in 2BUT. This is also evident from the protons in the aliphatic region which integrate to two protons each, contrary to the expected four protons. As for the central aromatic ring and the region from the (−CH_2_) in the picolinic arm to the pyridyl ring, integration data also showed symmetry as these protons only showed as one symmetric pair. An example is the central aromatic ring protons which integrate to one while there are two of each of their kind in the whole molecule.

After complexation, proton NMR was further applied in the characterization of the complexes. However, due to the poor solubility of the complexes, the spectral data were not good enough to be used to fully characterize the complexes. The complexes spectra were dominated by the solvent peaks which suppressed the analyte peaks. Therefore, other characterization techniques were used to conclusively characterize the complexes. The chemical shift data are displayed under Section 3.2 of this paper. In addition to the poor solubility of the complexes, the Ru(III) complex [Ru_2_(2BUT)(Cl)_6_] was also paramagnetic. This paramagnetism resulted in poor resolution of the multiplicity of the chemical shift of the Ru(III) complex and this was more observable in the aromatic region than in the aliphatic region. Using the aliphatic region, it was observed that the ligand had lost its planarity upon complexation with ruthenium. The chemical shift of the 2-picolinic arms of the free ligand was observed at 4.67 ppm, and upon complexation to Ru(III), they shifted to 2.60 ppm which indicated a loss of planarity. As for the Ru(II) complex [Ru_2_(2BUT)(DMF)_2_(DPA)](BH_4_)_4_, it also showed poor multiplicity, which could be attributed to poor solubility. However, some notable chemical shifts could be observed, for example, the dimethylformamide peaks were observed at chemical shifts of 0.81–0.85 ppm. In this complex, the planarity of the ligand was also lost, as is evident from the aliphatic protons which were observed in different chemical shifts as compared to the free ligand. Nonetheless, since the Ru(II) is diamagnetic, the chemistry around the aromatic region was detectable.

### 3.3. Antibacterial Activity

#### 3.3.1. MIC Assay

**Figure 4:**
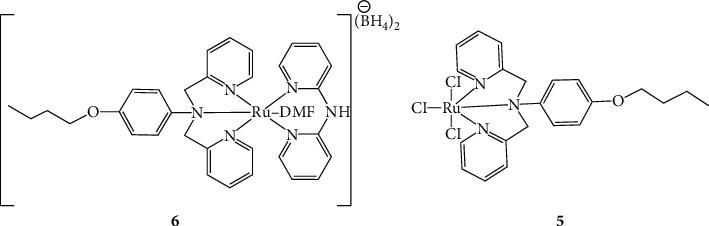
The mononuclear ruthenium (II/III) complexes reported in our previous work [6]. The difference with these analogues is that the complexes (**3** and **4**) in this paper are bridged by a four-member aliphatic chain. The chain in the mononuclear complexes does not connect to anything.

The synthesized ligand and the corresponding Ru(II/III) complexes were evaluated for their antibiotic properties against four bacterial species: *S. aureus, K. pneumoniae*, MRSA, and MDR *K. pneumoniae*. Following the lack of antibacterial activity of the complexes towards drug-resistant bacteria in our previous work [6], the modified analogue complexes reported herein were investigated for antibacterial activity towards the mentioned bacteria as in the previous work. We observed that the new analogues had broad-range antibacterial activity on both Gram-positive and Gram-negative non-drug-resistant bacteria. This was explained by the MIC data depicted in Table 5 and the disc diffusion assay data shown in Table 6. The MIC assay showed that there was comparatively more inhibitory activity towards the Gram-positive than the Gram-negative bacteria. As in our previous work, we suggest that this could be attributable to the differences in the Gram-negative bacterial cellular membranes compared to the Gram-positive, where the former uses a porin or efflux pumps in the cell membrane for ingestion and excretion. These porins may affect what enters the bacterial cells according to the size of the foreign object, and in this case, we suspected the compounds were affected by this [6, 11, 14]. All compounds had activity towards all the non-drug-resistant bacteria. However, the complexes had superior activity than the ligands. This is because the complexes had better activity at low concentrations, with the exception of the ligand 2BUT having exceptional potent activity towards *S. aureus.* Following the restructuring of the complexes in [6], the antibacterial activity was observed to be significantly different in the new analogues. The complexes in this work were of a dinuclear nature as compared to the mononuclear complexes in [6] as shown in Figure 4. Although dinuclear complexes have not been studied extensively compared to their mononuclear counterparts, they present interesting chemistry that has only been unveiled recently [12]. The superiority of dinuclear metal complexes has previously been explored in the context of potential anticancer and antimicrobial agents [13]. In the present study, it is likely that the dinuclear nature of the complexes favours their interaction with biological molecules to the detriment of bacterial cells. This is also suspected to be due to the dinuclear complexes having doubled the capabilities of the complexes on the activity.

The neutral complex **4** showed activity towards *S. aureus* and *K. pneumoniae*, and this was the case for the analogue complex **5** from our previous work. The dinuclear complex **4** showed better activity as compared to complex **5** where the MIC for *S. aureus* was 7.50 mg/mL compared to 10.00 mg/mL. However, the MIC for *K. pneumoniae* was 10.00 mg/mL for both complexes. The charged complex **3** in this work showed better activity compared to complex **6** from our previous paper. However, for *S. aureus*, similar activity was observed from the analogues. The dinuclear complex displayed better activity for *K. pneumoniae* as opposed to the mononuclear analogue. Interestingly, complex **3** had some activity towards MRSA as opposed to its analogue complex **6**. Also, the ligand DPA showed activity on MRSA. Complex **3** had bactericidal activity towards MRSA, which means that the complex was able to kill off the bacteria, while the DPA ligand had a bacteriostatic activity towards MRSA, implying that the ligand was able to stop the reproduction of the bacteria. Furthermore, the data indicated that the main ligand for complex **3**, 2BUT, still showed no activity towards MRSA. As for the observed activity of 2BUT on *S. aureus* and no activity on MRSA, it was inferred that this compound had a similar mode of action towards these two bacteria. These modified dinuclear complexes were suspected to possess better activity due to their structural characteristics. The ligands found in these complexes have planar aromatic rings (the pyridyl moieties and the aromatic ether-amine central ring) which may have induced better affinity to physically bind to biological molecules [15]. Furthermore, the dinuclear nature of complexes **3** and **4** may have greatly influenced their antibacterial properties. This is because the introduction of the less polar aliphatic chain bridge may have increased the lipophilicity of the ligands and ultimately the whole complex [16]. In that case, it would be possible that there was better interaction and easier access into the bacterial cells due to this phenomenon. Unfortunately, there was still no activity from all the compounds on MDR *K. pneumoniae*.

#### 3.3.2. Disc Diffusion Assay

After determining the lowest bioactive concentration of the compounds, disc diffusion was used to find the extent to which the bacterial colony growth is affected by the compounds. Just as in the MIC and even in the previous work's analogue complexes [6], there was an observed high activity of the complexes on the Gram-positive *S. aureus* as opposed to the Gram-negative *K. pneumoniae.* No activity was observed for MDR *K. pneumoniae* from all the compounds. However, in terms of MRSA, there was still some activity from complex **3** and the DPA ligand, just as seen on the MIC data. Furthermore, it was still interesting that there was no activity evident for complex **3**'s major ligand 2BUT and even its analogue complex **6** from the previous work. In that case, we suspect that, upon coordinating 2BUT with ruthenium, the 2BUT ligand became enabled or complex **2** which had no 2BUT ligand became enabled when coordinated with the 2BUT ligand, consistent with previous studies [17–20]. As for the analogue complex **6**, we suspected the introduction of the bridge in complex **3** may have induced the bioactivity of this complex towards MRSA. The zones of inhibition for all compounds are given in Table 6. Notably, 2BUT had some activity towards *S. aureus,* suggesting that the mode of action may be the same as that of MRSA, which may explain the lack of activity from this ligand on MRSA.

#### 3.3.3. DNA Chelation Assay

**Figure 5:**
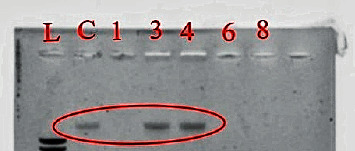
DNA cleavage analysis on 0.8% agarose gel. Lane L, 1 kb ladder; C, control DNA; 1, complex **2**; 3, BUT; 4, DPA; 6, complex **4**; 8, complex **3**. The gel indicates that all the complexes had DNA nuclease activity while the ligands did not cleave nor nuclease the DNA.

This assay was used to assess the DNA cleaving properties of the compounds. All the compounds which had activity towards *S. aureus* and MRSA were tested to see if they could cleave DNA into different forms. The DNA used had been freshly extracted from *S. aureus.*Figure 5 shows the interaction of these compounds with the DNA.

Lane L represents the DNA marker, while C is the untreated control DNA. Even though ligands showed good activity with the other assays, in this assay the ligands did not show any DNA cleavage. The 2BUT and DPA ligand are on lanes 3 and 4. These findings suggested that the 2BUT and DPA ligands had different modes of action contrary to the complexes. We concluded that the introduction of the metal centre to the ligands may induce more modes of action or disable the ligands, hence the observation on the gel. The complexes on lanes 1, 6, and 8 showed interesting results. Just as observed from our previous research [6], these complexes also had potent activity towards DNA. We noted that the complexes completely denatured the DNA. We had suggested and still suggest that the strong activity was due to the strong affinity of the complexes towards DNA. The labile ligands (chlorido and dimethylformamide), planar heterocyclic polypyridyl ligands, and the ruthenium metal centre are assumed to be the contributing factors to the observed DNA denaturing activity [6, 21–23]. These data suggest that DNA nuclease/denaturing may be the mode of action for the complexes, especially that they showed bactericidal concentrations.

## 4. Conclusions

It may be concluded that a new ligand 2BUT and 2 new complexes were successfully synthesized and characterized spectroscopically through FTIR, UV/Vis, elemental analysis, and magnetic susceptibility. The complexes UV/Vis data showed the energetically observable transitions associated with the complexes. The data suggested the complexes to adopt a low spin *d*^*5*^ and *d*^*6*^ Ru(II/III), respectively. Magnetic susceptibility studies also suggested the same spin for the Ru(II/III) complexes. FTIR showed all the complexes' important functional groups in the near IR. There was an observed decrease in some metal to ligand vibrational bands which indicated coordination of the metal to the ligands. Mass spectrometry and elemental analysis showed the compounds were successfully synthesized. Proton NMR showed that the ligand was successfully synthesized. However, the complex proton NMR was challenging to assign but did prove they were formed. The biological assays showed that complexes and ligands were indeed bioactive. Disc diffusion assay showed that there was more activity towards the Gram-positive bacteria. We also observed that there was more activity against the non-drug-resistant strains *S. aureus* and *K. pneumoniae.* There was some notable activity towards MRSA from complex **3** as opposed to the monometallic complex **6** in our previous work. The DPA ligand also showed some activity towards MRSA. However, all the complexes showed no activity towards MDR *K. pneumoniae* just like in the previous paper. DNA gel electrophoresis showed that the complexes had potent DNA chemical nuclease/denaturing; this was then suggested to be the mode of action of the complexes or one of the modes of action that could be observed from the complexes. Also, it was observed that the modifications of the analogues of these complexes in our previous work did indeed show some new activity for the complexes.

## Figures and Tables

**Table 1:** FTIR assignments for ligands and complexes.

Compound	C-O (cm^−1^)	Ar-N (cm^−1^)	C=C (cm^−1^)	C-H (cm^−1^)	N-H (cm^−1^)
DPA	—	1477	1529	3019	3179–3253
2BUT **(1)**	1230	1476	1508	2926	—
[Ru_2_(2BUT)(DMF)_2_(DPA)](BH_4_)_4_**(3)**	1244	1434	1504	2961	—
[Ru_2_(2BUT)(Cl_6_] **(4)**	1245	1435	1508	2948	—

The vibrational spectroscopic data indicated all the functional groups for the ligands and the complexes. Also, it was seen that the ligand functional groups bound by the metal demonstrated a shift in the vibrational frequency, and this indicated that there is coordination.

**Table 2:** Complexes and their associated electronic spectroscopy bands.

Transitions	[Ru_2_(2BUT)(DMF)_2_(DPA)](BH_4_)_4_**(3)** (nm) (*ε*×10^4^(L mol^−1^ cm^−1^)) (0.32 mM)	[Ru_2_(2BUT)(Cl)_6_] **(4)** (nm) (*ε*×10^4^(L mol^−1^ cm^−1^)) (0.62 mM)
*π*-*π*^*∗*^ and CT	<**262** [1.009], **345** [0.5469]	<**297** [5.240]
*d-d*	**462** [0.1626], **532** [0.1325]	**412** [0.1356], **604** [0.0750]

CT: charge transfer bands. The electronic spectral data indicated the complexes are low spin Ru(III) *d*^*5*^ and Ru(II) *d*^*6*^ complexes. Also, the molar absorptivity of the complexes' bands demonstrates that they are their true assigned transitions.

**Table 3:** Experimental and calculated elemental analysis of complexes.

Compound molecular formula (according to EA data)	Experimental	Calculated
	C: H: N	C: H: N
[Ru_2_(2BUT)(DMF)_2_(DPA)](BH_4_)_4_] •0.9H_2_O	56.89 : 6.33 : 13.59	56.5 : 6.45 : 13.98
[Ru_2_(2BUT)(Cl)_6_] •0.6H_2_O	45.66 : 3.70 : 7.50	45.26 : 3.9 : 7.92

Analytical data indicate the complexes only had impurity of water. The data also showed that the complexes' empirical formula is similar to the calculated data with very low differences.

**Table 4:** Experimental and calculated mass analysis of complexes.

Compounds	Adduct	Experimental	Calculated
C_40_H_41_N_6_O_2_**(1)**	[M+H]^+^	637.3412	637.8075
C_66_H_72_N_14_O_4_Ru_2_**(3)**	[M+2Na-H]^+^	1372.7879	1373.3612
C_41_H_43_Cl_6_N_6_O_2_Ru_2_**(4)**	[M+K]^+^	1104.5452	1103.9332

The mass spectrometric data indicate successful synthesis of the compounds. All the compounds were run on a positive ion mode with molecular ion peaks being observed with proton and metal adducts.

**Table 5:** MIC of all active compounds.

Microbe	Li[Ru(Cl)_4_(DPA) **(2)** (mg/mL)	[Ru_2_(2BUT)(DMF)_2_(DPA)](BH_4_)_4_] **(3)** (mg/mL)	[Ru_2_(2BUT)(Cl)_6_] **(4)** (mg/mL)	2BUT **(1)** (mg/mL)	DPA (mg/mL)
*S. aureus*	5.00	1.88	7.50	0.03	15.00
MRSA	0	2.50	0	0	15.00
*K. pneumoniae*	10.00	7.50	10.00	20.00	1.88
MDR *K. pneumoniae*	0	0	0	0	0

Note: 0: not active. The compounds were assayed for their MIC towards *S. aureus, K. pneumoniae*, MRSA, and MDR *K. pneumoniae*. The assay revealed all complexes to display activity towards all non-drug-resistant bacteria. Only [Ru_2_(2BUT)(DMF)_2_(DPA)](BH_4_)_4_] complex displayed activity on MRSA together with the ligands. However, no compound indicated activity towards MDR *K. pneumoniae.* After this, the complexes were assayed for their bacterial growth inhibition using disc diffusion assay.

**Table 6:** Zones of inhibition of all compounds.

Microbe	Li[Ru(Cl)_4_(DPA) **(2)** (mm)	[Ru_2_(2BUT)(DMF)_2_(DPA)](BH_4_)_4_] **(3)** (mm)	[Ru_2_(2BUT)(Cl)_6_] **(4)** (mm)	2BUT **(1)** (mm)	DPA (mm)
*S. aureus*	9	8	8	8	9
MRSA	0	8	0	0	7
*K. pneumoniae*	7	7	7	7	8
MDR *K. pneumoniae*	0	0	0	0	0

The disc diffusion assay was used to the ligands and complexes for their activity towards the three bacterial organisms *S. aureus, K. pneumoniae*, MRSA, and MDR *K. pneumoniae*. This was done after their MIC values were assayed to assess the extent to which the compounds inhibit the growth of the bacteria. Just as in the MIC assay, all the compounds indicated activity towards all non-drug-resistant bacteria while [Ru_2_(2BUT)(DMF)_2_(DPA)](BH_4_)_4_], [Ru_2_(2BUT)(Cl)_6_], 2BUT, and DPA had activity towards MRSA. No compounds showed activity on MDR *K. pneumoniae.*

## Data Availability

The characterization and biological assay data used to support the findings of this study are included within the article, summarized in tables and under materials and methods. However, there is a supplementary material data sheet that includes the spectral data of the compounds from Figure S1 to Figure S13.

## Supplementary Materials

Supplementary Materials Figure S1: proton NMR spectrum of 2BUT **1**. Figure S2: FTIR spectrum of 2BUT **1**. Figure S3: HRESI-MS spectrum of 2BUT **1**. Figure S4: FTIR spectrum of complex **3**. Figure S5: UV/Vis spectrum of complex **3**. Figure S6: HRMS spectrum of complex **3**. Figure S7: HRMS enlarged spectrum of complex **3** at assigned molecular ion *m*/*z* 1372.7879. Figure S8: proton NMR of Ru(II) complex **3**. Figure S9: FTIR spectrum of complex **4**. Figure S10: UV/Vis spectrum of complex **4**. Figure S11: HRMS spectrum of complex **4**. Figure S12: HRMS enlarged spectrum of complex **4** at assigned molecular ion *m*/*z* 1104.5452. Figure S13: proton NMR spectrum of the Ru(III) complex **4** ().

## References

[1] Southam H. M., Butler J. A., Chapman J. A., Poole R. K. (2017). The microbiology of ruthenium complexes. *Advances in Microbial Physiology*.

[2] Reedijk B. J. (2008). Metal-ligand exchange kinetics in platinum and ruthenium complexes. *Pltinum Metals Review*.

[3] Abebe A., Hailemariam T. (2016). Synthesis and assessment of antibacterial activities of ruthenium (III) mixed ligand complexes containing 1, 10-phenanthroline and guanide. *Bioinorganic Chemistry and Applications*.

[4] Gopinath K., Karthika V., Gowri S., Senthilkumar V., Kumaresan S., Arumugam A. (2014). Antibacterial activity of ruthenium nanoparticles synthesized using Gloriosa superba L. leaf extract. *Journal of Nanostructure in Chemistry*.

[5] Lam P.-L., Lu G.-L., Hon K.-M. (2014). Development of ruthenium (II) complexes as topical antibiotics against methicillin resistant *Staphylococcus aureus*. *Dalton Transactions*.

[6] Matshwele J. T., Nareetsile F. M., Mapolelo D. T. (2020). Synthesis of mixed ligand ruthenium (II/III) complexes and their antibacterial evaluation on drug-resistant bacterial organisms. *Journal of Chemistry*.

[7] Lalitha K. (2004). Manual on antimicrobial susceptibility testing:,” Performance standards for antimicrobial testing. *Twelfth Informational Supplement*.

[8] Monteiro M. C. R., Nascimento F. B., Valle E. M. A. (2010). Experimental and theoretical study of the kinetics of dissociation in cis-[RuCl2(P-P)(N-N)] type complexes. *Journal of the Brazilian Chemical Society*.

[9] Halcrow M. A. (2013). Jahn-Teller distortions in transition metal compounds, and their importance in functional molecular and inorganic materials. *Chemical Society Reviews*.

[10] Willock D. (2009). *Molecular Symmetry*.

[11] Weseler A., Geiss H. K., Saller R., Reichling J. (2005). A novel colorimetric broth microdilution method to determine the minimum inhibitory concentration (MIC) of antibiotics and essential oils against Helicobacter pylori. *Ie Pharmazie-An International Journal of Pharmaceutical Sciences*.

[12] Li G., Zhu D., Wang X., Su Z., Bryce M. R. (2020). Dinuclear metal complexes: multifunctional properties and applications. *Chemical Society Reviews*.

[13] Li F., Feterl M., Warner J. M., Keene F. R., Collins J. G. (2013). Dinuclear polypyridylruthenium (II) complexes: flow cytometry studies of their accumulation in bacteria and the effect on the bacterial membrane. *Journal of Antimicrobial Chemotherapy*.

[14] Martínez J. L., Baquero F. (2014). Emergence and spread of antibiotic resistance: setting a parameter space. *Upsala Journal of Medical Sciences*.

[15] Claudel M., Schwarte J. V., Fromm K. M. (2020). New antimicrobial strategies based on metal complexes. *Chemistry*.

[16] Yang Y., Liao G., Fu C. (2018). Recent advances on octahedral polypyridyl ruthenium (II) complexes as antimicrobial agents. *Polymers*.

[17] Barai H. R., Lee D. J., Han S. W., Jang Y. J. (2016). *Interaction and Binding Modes of bis-Ruthenium (II) Complex to Synthetic DNAs*.

[18] Li F., Collins J. G., Keene F. R., Keene F. R. (2015). Ruthenium complexes as antimicrobial agents. *Chemical Society Reviews*.

[19] Manoharan P. T., Mehrotra P. K., Taquikhan M. M., Andal R. K. (1973). Optical and magnetic properties and geometry of some d5 ruthenium complexes. *Inorganic Chemistry*.

[20] Bryant G., Fergusson J., Powell H. (1971). Charge-transfer and intraligand electronic spectra of bipyridyl complexes of iron, ruthenium, and osmium. I. Bivalent complexes. *Australian Journal of Chemistry*.

[21] Sindhu Y., Athira C. J., Sujamol M. S., Joseyphus R. S., Mohanan K., Synthesis “ (2013). Synthesis, characterization, DNA cleavage, and antimicrobial studies of some transition metal complexes with a novel schiff base derived from 2-aminopyrimidine. *Synthesis and Reactivity in Inorganic, Metal-Organic, and Nano-Metal Chemistry*.

[22] Brabec V., Pracharova J., Stepankova J., Sadler P. J., Kasparkova J. (2016). Photo-induced DNA cleavage and cytotoxicity of a ruthenium (II) arene anticancer complex. *Journal of Inorganic Biochemistry*.

[23] Griffith C., Dayoub A. S., Jaranatne T. (2017). Cellular and cell-free studies of catalytic DNA cleavage by ruthenium polypyridyl complexes containing redox-active intercalating ligands. *Chemical Science*.

