# Supplementary material for: Antibacterial Activity of 2-Picolyl-polypyridyl-Based Ruthenium (II/III) Complexes on Non-Drug-Resistant and Drug-Resistant Bacteria
Source: Bioinorg Chem Appl. 2021 May 20;2021:5563209. doi: 10.1155/2021/5563209 (PMC8163554; doi:10.1155/2021/5563209)
Supplement: Supplementary Materials — Figure S1: proton NMR spectrum of 2BUT 1. Figure S2: FTIR spectrum of 2BUT 1. Figure S3: HRESI-MS spectrum of 2BUT 1. Figure S4: FTIR spectrum of complex 3. Figure S5: UV/Vis spectrum of complex 3. Figure S6: HRMS spectrum of complex 3. Figure S7: HRMS enlarged spectrum of complex 3 at assigned molecular ion m/z 1372.7879. Figure S8: proton NMR of Ru(II) complex 3. Figure S9: FTIR spectrum of complex 4. Figure S10: UV/Vis spectrum of complex 4. Figure S11: HRMS spectrum of complex 4. Figure S12: HRMS enlarged spectrum of complex 4 at assigned molecular ion m/z 1104.5452. Figure S13: proton NMR spectrum of the Ru(III) complex 4 (). [file 5563209.f1.docx]

**Supplementary Information**

**Antibacterial activity of 2-picolyl-polypyridyl-based ruthenium (II/III) complexes on non-drug resistant and drug resistant bacteria**

James T.P. Matshwele^1,3^, Sebusi Odisitse^1^, Daphne Mapolelo^2^, Melvin Leteane^2^, Lebogang G. Julius ^2^, David O. Nkwe^4^, Florence Nareetsile^2*^.

^1^Botswana International University of Science and Technology, Department of Chemistry and Forensic Sciences, Private Bag 16, Palapye, Botswana.
^2^University of Botswana, Faculty of Science, Private Bag 0704, Gaborone, Botswana.

^3^Botho University, Department of Applied Sciences, Gaborone, Botswana, PO Box 501564, Gaborone, Botswana.

^4^Botswana International University of Science and Technology, Department of Biological Sciences and Biotechnology, Private Bag 16, Palapye, Botswana.

Corresponding author: Florence Nareetsile; nareetsilef@ub.ac.bw

**Table of Contents**

[1. (4,4'-(butane-1,4-diylbis(oxy))bis(N,N-bis(pyridin-2-ylmethyl)aniline) (2BUT) (1) 2](#_Toc53773905)

[2. [Ru_2_(2BUT)(DMF)_2_(DPA)](BH_4_)_4_(3) 4](#_Toc53773906)

[3. [Ru_2_(2BUT)(Cl)_6_] •H_2_O (4) 7](#_Toc53773907)

# (4,4'-(butane-1,4-diylbis(oxy))bis(N,N-bis(pyridin-2-ylmethyl)aniline) (2BUT) (1)

Yield: 280 mg, 76.0%,. mp 145-147°C. IR (υmax /cm-1) (C-H) 2926, (C=C) 1508, (Ar-N) 1431.3, (C-O) 1230. ^1^H NMR (600 MHz, CDCl3, δ) 8.48 (m, 4H), 7.53 (m, 4H), 7.24 (m, 4H), 7.07 (m, 4H), 6.68-6.53 (dd, J = 8.6 Hz, 4H), 4.67 (s, 8H), 3.87 – 3.81 (t, 2H), 1.85 – 1.73 (q, 2H). HRESI-MS [M+H]+ m/z 637.3412 (calcd for C40H41N6O2 637.8075).

**Figure S1**: Proton NMR spectrum of 2BUT **1**

**Figure S2**:FTIR spectrum of 2BUT **1**

**Figure S3**: HRESI-MS spectrum of 2BUT **1**

# [Ru_2_(2BUT)(DMF)_2_(DPA)](BH_4_)_4_(3)

Yield: 160 mg, 62.0 %. IR (υ_max_ /cm^-1^) (C-H) 2961 (C=C) 1504, (C-O) 1244, (Ar-N) 1434.2, (C=O_DMF_) 1668. ^1^H NMR (600 MHz, CDCl_3_) δ 9.84 – 9.66 (m, 4H), 8.52 (m, 4H), 7.83 (m, 4H), 7.68 (m, 2H), 7.58 – 7.43 (m, 8H), 7.20 – 7.05 (m, 8H), 6.71 (m, 4H), 6.59 (m, 4H), 4.53 (s, 4H), 3.86 (m, 4H), 2.55 (m, 4H), 0.92 – 0.71 (m, 12H). UV-Vis (DMF; λmax [nm]): 262, 462, 532. HRESI-MS [M+2Na-H]+ m/z 1372.7879 (calcd for C_66_H_72_N_14_O_4_Ru_2_ 1373.3612)~~.~~ Anal. Found (%) for C_66_H_88_B_4_N_14_O_4_Ru_2_ **•**0.9H_2_O: 56.89 %; H, 6.33 %; N, 13.59 %. Calcd (%) C, 56.50 %; H, 6.45 %; N, 13.98 %.

**Figure S4**: FTIR Spectrum of complex **3**

**Figure S5**: UV/Vis Spectrum of complex **3**


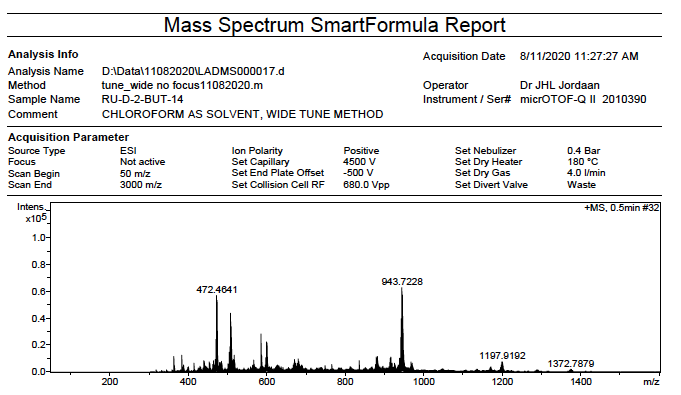


**Figure S6**: HRMS Spectrum of complex 3


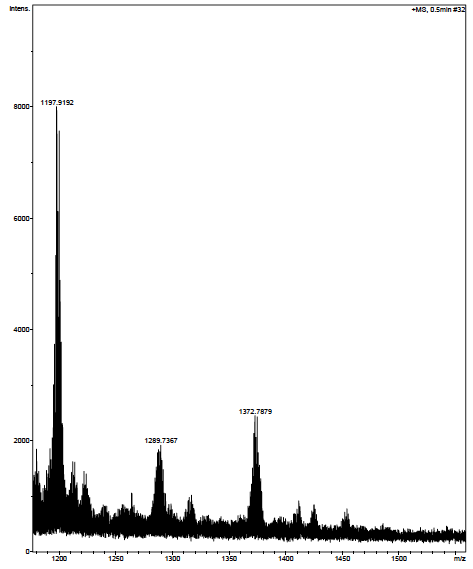


**Figure S7**: HRMS Enlarged spectrum of complex 3 at assigned molecular ion m/z 1372.7879

**Figure S8**: Proton NMR of Ru(II) complex 3

# [Ru_2_(2BUT)(Cl)_6_] (4)

Yield: 1700 mg, 70.0 %. IR (υ_max_ /cm^-1^) (H_2_O) 3403, (C-H) 2948, (C=C) 1508, (C-O) 1245, (Ar-N) 1435. ^1^H NMR (600 MHz, CDCl_3_) δ 7.46 -8.06 (m, 24H), 3.76 (t, *J* = 6.4 Hz, 4H), 2.60 (s, 8H), 1.89 – 1.85 (m, 4H). UV-Vis (DMF; λmax [nm]): 297, 412, 604. HRESI-MS [M+K]^+^ m/z 1104.5452 (calcd for C_41_H_43_Cl_6_N_6_O_2_Ru_2_ 1103.9332). Anal. Found (%) for C_41_H_43_Cl_6_N_6_O_2_Ru_2_ **•**0.6H_2_O: 45.66 %; H, 3.70 %; N, 7.50 %. Calcd (%) C, 45.26 %; H, 3.90 %; N, 7.92 %.

**Figure S9**: FTIR Spectrum of complex **4**

**Figure S10**: UV/Vis Spectrum of complex **4**


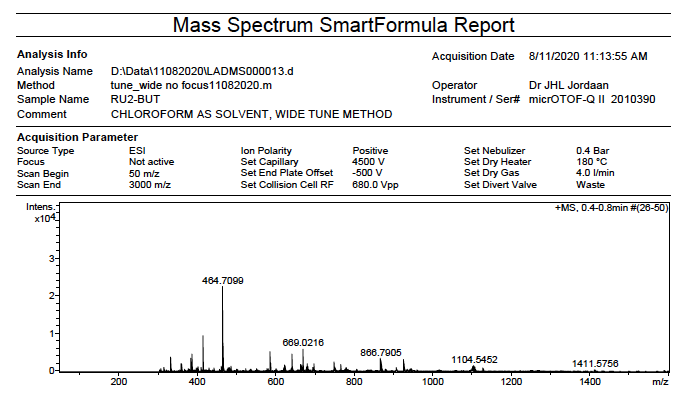


**Figure S11**: HRMS Spectrum of complex 4


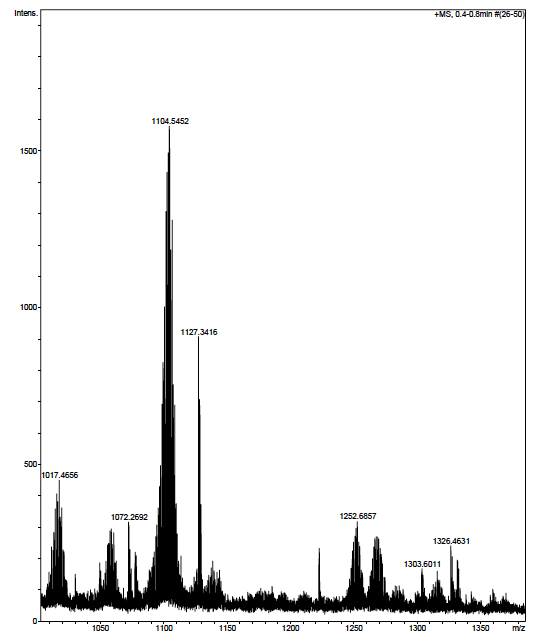


**Figure S12**: HRMS Enlarged spectrum of complex 4 at assigned molecular ion m/z 1104.5452

**Figure S13**: Proton NMR spectrum of the Ru(III) complex 4
